# Health Care Professionals’ Perspectives on the Secondary Use of Health Records to Improve Quality and Safety of Care in England: Qualitative Study 

**DOI:** 10.2196/14135

**Published:** 2019-09-26

**Authors:** Ana Luísa Neves, Dilkushi Poovendran, Lisa Freise, Saira Ghafur, Kelsey Flott, Ara Darzi, Erik K Mayer

**Affiliations:** 1 Patient Safety Translational Research Centre Institute of Global Health Innovation Imperial College London London United Kingdom; 2 Center for Health Technology and Services Research / Department of Community Medicine, Health Information and Decision Faculty of Medicine University of Porto Porto Portugal; 3 Centre for Health Policy Institute of Global Health Innovation Imperial College London London United Kingdom

**Keywords:** electronic health records, information technology, health policy, safety culture

## Abstract

**Background:**

Health care professionals (HCPs) are often patients’ first point of contact in what concerns the communication of the purposes, benefits, and risks of sharing electronic health records (EHRs) for nondirect care purposes. Their engagement is fundamental to ensure patients’ buy-in and a successful implementation of health care data sharing schemes. However, their views on this subject are seldom evaluated.

**Objective:**

This study aimed to explore HCPs’ perspectives on the secondary uses of health care data in England. Specifically, we aimed to assess their knowledge on its purposes and the main concerns about data sharing processes.

**Methods:**

A total of 30 interviews were conducted between March 27, 2017, and April 7, 2017, using a Web-based interview platform and following a topic guide with open-ended questions. The participants represented a variety of geographic locations across England (London, West Midlands, East of England, North East England, and Yorkshire and the Humber), covering both primary and secondary care services. The transcripts were compiled verbatim and systematically reviewed by 2 independent reviewers using the framework analysis method to identify emerging themes.

**Results:**

HCPs were knowledgeable about the possible secondary uses of data and highlighted its importance for patient profiling and tailored care, research, quality assurance, public health, and service delivery planning purposes. Main concerns toward data sharing included data accuracy, patients’ willingness to share their records, challenges on obtaining free and informed consent, data security, lack of adequacy or understanding of current policies, and potential patient exposure and exploitation.

**Conclusions:**

These results suggest a high level of HCPs’ understanding about the purposes of data sharing for secondary purposes; however, some concerns still remain. A better understanding of HCPs’ knowledge and concerns could inform national communication policies and improve tailoring to maximize efficiency and improve patients’ buy-in.

## Introduction

### Background

The use of electronic health records (EHRs) for secondary purposes, such as health research, public health surveillance, quality improvement, and safety monitoring, is essential for improving patient care [1]. In recent years, the exponential growth in EHR adoption, together with developments in health care informatics and data mining tools, has generated a range of health discoveries that ultimately improve the quality and safety of health care delivery [2-4]. Furthermore, health care data sharing for secondary uses, and particularly for research purposes, seems to be supported by the wider public [5-7]. At the same time, evidence suggests that harm due to inappropriate or insufficient use of available data is a real problem with potential serious consequences such as increased mortality and financial burden [8]. Therefore, while preserving patients’ privacy, health care systems must find solutions to leverage health care data to deliver safer and better care.

Governmental initiatives worldwide have been advocating the use of EHR for secondary purposes. In the United States, the Health Information Technology for Economic and Clinical Health Act (2009) sets the adoption and meaningful use of EHR as a key national goal [9]. According to this Act, as a condition for clinicians and hospitals to receive incentive payments, they needed to *meaningfully use* certified EHRs. This refers to the use of EHR data to inform quality improvements, monitor safety, and drive efficiency. It can also refer to much broader societal health efforts such as reducing health disparities, engaging patients and families in their health, and improving care coordination and data security [10]. In Europe, the European Institute for Innovation through Health Data was launched with the aim to develop and promote best practices in the governance, quality, semantic interoperability, and uses of EHR data, including, importantly, its reuse for research purposes [11].

In the United Kingdom, the National Health Service (NHS) Care.data also aimed to securely link information from general practitioners’ (GPs) records with data from secondary care, with the ultimate goal of providing a better understanding of patients’ holistic needs [12]. However, the progress of this program stalled for a variety of reasons, including general concerns about data security, lack of information about benefits of data sharing, and complications around opt-out procedures, which eventually lead to the closure of the program in 2016 [13]. This experience showed that although governmental endorsement is key, it is not per se enough to ensure a successful implementation. Lessons learned include a greater awareness of the importance of adequately engaging with patients, the wider public, and, importantly, health care professionals (HCPs) in the debate about health care data sharing to learn their experiences and manage governmental programs accordingly [14].

Being in the frontline of health care delivery, HCP are often patients’ first and closest point of contact in what concerns the communication of the purposes, benefits, and risks of sharing health care data for secondary uses. The provider-patient relationship in health care is a paramount example of a *trust relationship*; the nature of this trust-based relationship is the key reason why their support and engagement is fundamental to ensure patients’ buy-in [15]. Provider-based communication with patients about the importance of using health care data for secondary purposes is critical: it enhances patients’ awareness of the benefits of sharing their health information, increases patients’ buy-in, and can ultimately have a positive impact on the availability of data for these uses. Altogether, these reasons justify the vital role of HCPs on the successful implementation of programs aiming to implement, or enhance, health care data sharing for secondary purposes. However, to date, most research in the United Kingdom focused on patients’ perspectives [6,16], and the perceptions of HCPs on data sharing for these purposes were seldom evaluated.

### Objectives

This study aimed to assess HCPs’ perspectives on the secondary uses of health care data and, specifically, explore their knowledge on its purposes and the main concerns about data sharing processes.

## Methods

### Overview of the Methods Used

To meet the study’s aims and objectives, a qualitative descriptive approach was adopted. In-depth interviews were chosen because of their ability to capture rich, descriptive data about individual perception, attitudes, and behaviors [17]. A multidisciplinary team including medical doctors (ALN, EKM, SG, and AD), a neuroscientist (DP), a health service researcher (KF), and a cognitive scientist (LF) with previous experience in qualitative research performed this study.

### Recruitment

HCPs from a variety of roles (ie, doctor, nurse, and allied HCP) were invited to participate by email, using a combination of recruitment approaches, whereas primary and secondary care doctors were invited through UK’s largest professional network of doctors (Doctors.net) membership, and HCPs from other roles were recruited from medeConnect Healthcare Insight’s contact network database. Purposive sampling by health care role was used, and participants from mixed roles were included. Participants were excluded if they were previously involved in clinical research (ie, having treated or managed patients who had participated in clinical trial or being a principal or site investigator for clinical trials). Informed consent was obtained for each participant when replying to a screener survey.

### Data Collection

A total of 30 Web-based in-depth interviews were conducted between March 27, 2017, and April 7, 2017. In-depth interviews allow the interviewer to explore and examine a given topic or experience in detail and are therefore an effective method for interpretative inquiry [18].

A Web-based bulletin system provided by medeConnect Healthcare Insight was used. A topic guide with open-ended questions was used to cover all the relevant topics during the online interviews, particularly their knowledge on the purposes of secondary uses of health care data, as well as their main concerns about data sharing processes (Multimedia Appendix 1). Questions were launched over the course of several days so that participants had time to reflect and answer each question.

As participants typed their own answers in the online bulletin system, transcription was automatic. The transcripts were compiled verbatim, following the required reliability and validity procedures for qualitative studies, and were not returned to participants for comments and/or corrections. The online interviews were hosted on secure servers belonging to the software providers for the interview software.

All interviews were conducted in English, and the interviewer had no established relationship with participants before study commencement. As online interviews were used, only the participants and the interviewer were present. Participants had a minimal knowledge of the characteristics of the interviewer and of the research team; thus, the potential for bias and assumptions was kept to the minimum. No repeat interviews were conducted.

### Data Analysis

A total of 2 independent researchers systematically reviewed the transcripts, using the framework analysis method, which includes 5 main stages: familiarization, identifying a thematic framework, indexing, charting, and mapping and interpretation [19]. The charting stage is applied as a principle for developing the coding framework through a process of abstraction to ensure that coding elements that might have been missed with an a priori approach are adequately captured [19]. The defining feature of this method is the organization of qualitative data as a matrix output: rows (ie, cases and interviewees), columns (ie, themes), and cells of summarized data, thus providing a structure that systematically reduces qualitative data to analyze it by theme [20].

At every stage of the data analysis process, the coding framework was kept deductive and inductive, allowing the ongoing inclusion of emergent themes. Themes were supported by quotations derived from the interviews. Data saturation was reached after 22 interviews. As participants did not provide consent for further contact, it was not possible to ask them to provide feedback on the findings. The findings will be shared with patient partners (Research Partners Group), who will be involved in the codevelopment of a dissemination strategy and in summarizing the research findings into lay summaries and reports. The Consolidated Criteria for Reporting Qualitative studies were used to ensure the study meets the recommended standards of qualitative data reporting (Multimedia Appendix 2).

## Results

### Participants’ Characteristics

The 30 HCPs who completed the interview represented a variety of geographic locations across England (London, n=12; West Midlands, n=6; East of England, n=4; North East England, n=4; and Yorkshire and the Humber, n=2), covering both primary (n=11) and secondary care services (n=17). Of the 30 participants, 2 did not provide geographic location and care setting information. A full description of the participants by professional role is shown in Table 1.

Participants identified a wide range of individuals and organizations with whom they share EHR data (Table 2). Respondents acknowledged to share health care data with a variety of individuals and organizations for secondary purposes, including administrative and finance departments, occupational health and public health services (ie, Public Health England), health care trusts and commissioners, and teams involved in evaluation and research (Table 2).

**Table 1:** Description of participants by professional role.

Role		Value, n (%)
**Primary care**		
	GP^a^	5 (17)
	GP receptionist	3 (10)
	Practice manager	3 (10)
**Secondary care**		
	Allied health professional	9 (30)
	Consultant	5 (17)
	Pharmacist	1 (3)
	Specialist nurse	2 (7)
Unknown		2 (7)

^a^GP: general practitioner.

**Table 2:** Health care professionals’ perceptions on which individuals and organizations have access to health care data.

Parties with access to health care data		Examples
**Health care professionals (clinical staff)**		
	Primary care	General practitioners, other local staff within primary care teams, and dentists
	Secondary care	Hospital staff and community care
	Tertiary care	Hospital staff and voluntary sector organizations
**Secondary uses**		
	Occupational and public health	Occupational health departments; Public Health England
	Trusts and commissioners	Trusts, Clinical Commissioning Groups, and National Health Service England
	Evaluation and research	Audit teams and monitoring systems; research teams
	Other	Judiciary system (courts and police) and schools
Supporting services		Biochemistry services, pathology services, suppliers and equipment companies, administrators, finance departments, and information technology

### Understanding of the Purposes of Sharing Health Care Data for Secondary Uses

The level of content and details varied greatly between interviews; however, all participants were able to identify at least one secondary purpose for health care data sharing. Thematic analysis of the patients’ narratives revealed 5 emerging themes, including (1) patient profiling, (2) research and evidence-based practice, (3) quality assurance, (4) public health purposes, and (5) health care delivery planning (Textbox 1).

Use of health care data to profile patients and improve tailored care appeared as a relevant purpose for several participants. HCPs highlight that analyzing health care data can provide useful insights into the context of preventive medicine, identify high-risk groups, and inform the design and implementation of tailored preventive measures (Textbox 1).

A few participants mentioned the importance of sharing health care data with researchers to generate evidence to support, guide, and improve health care delivery (Textbox 1). Participants also acknowledged quality assurance as a major purpose of sharing health care data for purposes beyond individual care. Pragmatic examples included the use of health care data to quantify and monitor various aspects of quality of care (ie, effectiveness and timeliness of service delivery) and to evaluate the compliance with previously set quality standards (Textbox 1).

Sharing health care data for public health purposes was also mentioned as a means to provide insights on disease surveillance and outbreaks. In this context, 2 HCPs recognized that health care data can be particularly useful to identify geographic trends and thus expose underserved or excluded areas or groups of individuals (Textbox 1).

Finally, HCPs emphasized that sharing EHR with trusts and commissioners can support planning and optimization of health care delivery (Textbox 1). Participants mention that these data can inform resource allocation and service provision and contribute to the improvement of current pathways for patient care and to the development of new ones.

### Concerns Regarding Electronic Health Record Data Sharing Beyond Individual Care

Most participants declared feeling comfortable with data sharing policies and their implementation. One participant highlighted the influence of having personally experienced the benefits of data sharing and how this positively impacted his perceptions:

**Understanding of purposes of secondary uses of health care data and thematic analysis of the patients’ narratives revealing 5 emerging themes.**

**Theme 1: patient profiling**

- I would imagine [data sharing] is useful for creating key profiles for injury types, mechanisms, recovery and therefore contributing to strategies for prevention of such injuries to others. [ID 8]

**Theme 2: research and evidence-based practice**

- Being an allied health professional, many if not all our treatments are guided by research which is essential for effective management. For this reason it would be important to share patient data. [ID 18]
- Well planned effective research supports new methods of treatment. [ID 11]

**Theme 3: quality assurance**

- I think it is important to share information on service performance to ensure that services are functioning effectively in treatment delivery and identifying areas of good or bad treatment delivery. [ID 11]
- […] previously [collected] data contribute to seeing if quality standards were being met in GP setting, such as chronic disease monitoring. [ID 28]
- Sharing patient information beyond patient individual care for research or statistical purposes, [allows] to check if care services provided to patient are good and if patient receive them at the right time. [ID 16]
- […] a lot of other information is used for audit and service improvement it uses patient identifiable data but is always anonymised before going anywhere else. [ID 5]

**Theme 4: Public health purposes**

- I feel I have some understanding when patients’ data is used for individual and non-individual care with other organisations such as area data when looking at epidemics. [ID 13]
- Data sharing can help to understand practice variations around the country or in different areas of the organization. [ID 9]
- […] you can also then use the data to compare how your area is doing compared to the national average. [ID 12]

**Theme 5: Health care delivery planning**

- [data sharing could] allow resource allocation and service provision from health care providers. [ID 28]
- I definitely think there is a place for patient data sharing when planning future services because it could improve services provided to patients. Additionally, it could allow more effective strategies to be put into place. [ID 18]
- Use of personal information allows for the amendment of and planning of new pathways for patient care. [ID 1]
- For example: Patient A might have followed a distinct pathway for a medical condition that was found to be too unwieldy and investigations might have taken place too late in the pathway. By sharing specific information about patients then the pathways can be reviewed and amended and improved for others in the future. [ID 1]

Nevertheless, some concerns were also mentioned. The themes that emerged formed 6 identifiable but sometimes interwoven concerns: (1) data inaccuracy, (2) patient unwillingness to share their data, (3) challenges to obtain free and informed consent, (4) data security, (5) lack of adequacy/need for clear policies, and (6) potential patient exposure to distress/exploitation (Textbox 2). Regarding data inaccuracy, a few participants mentioned being concerned about its implications on both diagnostic characterization and evaluation of Quality and Outcomes Framework targets (Textbox 2). Some participants also highlighted being concerned that patients might not be able to understand current communication policies around data sharing for secondary purposes and therefore perceive it as a negative initiative and thus decide to opt-out (Textbox 2). Respondents indicated that obtaining patients’ free and informed consent to share their data may pose particular challenges, as patients may feel external pressure to share their information and therefore not take this decision as freely as would be desirable. One participant also mentioned being concerned about the adequacy and completeness of the information provided to patients when asking for their consent to share data, particularly when consent is obtained verbally (Textbox 2). Several participants reported having concerns on the security of data transmission and, importantly, about the possibility of data being inappropriately abusively assessed by third parties during this process (Textbox 2).

Participants reported concerns not only about the robustness of regulations and policies but also about the ways these are communicated to the public, which may create a negative impact on patients’ buy-in (Textbox 2).

Altogether, the themes mentioned have the potential to result in deleterious consequences for the patient and thus to contribute to patient’s exposure to distress/possible exploitation (Textbox 2).

**Concerns regarding health care data sharing beyond individual care and thematic analysis of the patients’ narratives revealing 6 emerging themes.**

**Theme 1: data inaccuracy**

- I […] have concerns about data accuracy and implications of diagnostic categorization, and particular concerns regarding data recording and its implications on QOF DATA and targets. [ID 22]

**Theme 2: patients’ unwillingness to share data**

- I am concerned patients […] [can] feel that sharing data is a negative thing and a breach of confidentiality.

**Theme 3: quality assurance**

- I am happy to share where I have consent, though I am not entirely convinced that the patient understands the different scenarios. [ID 1]
- I have concerns that there is pressure for me to ask patients and their carers to agree for their information to be shared for research purposes. I believe that research […] is essential to try to find curative treatments however I believe that the patient has the right to say no. [ID 3]
- Concerns about formally getting consent from the patients especially regarding the equipment company, it is always verbal so we have no evidence of what was said and what the patient said. Is it truly informed, have we given the correct information? [ID 12]

**Theme 4: data security**

- The only concern I have is regarding the security of the information that is shared electronically. Are IT processes to safeguard data robust, considering occasional reports in local media of leaks of personal data from private companies? [ID 6]
- I always remain concerned about how safe this data remains during transmission especially. [ID 9]
- I am worried hackers and people not delivering care to an individual, should not be able to access this sensitive information about someone's health. [ID 28]

**Theme 5: lack of adequacy/understanding of current policies**

- I would hope that the sharing of data for secondary use is strictly regulated, confidential and, where necessary, anonymous. [ID 14]
- I am afraid that policies are inadequate regarding data security. [ID 22]
- I am cynical about Government’s ability, and track record, on protecting data. [ID 27]
- I am concerned that the policies can sometimes be too detailed for staff/patients to fully understand. [ID 10]

**Theme 6: patient exposure to distress/possible exploitation**

- Not specifically, but one is always mindful of the possibility of abuse of access. [ID 20]
- The concerns are often about the patient being vulnerable and giving out information that could lead to them becoming distressed/possible exploitation. [ID 29]

## Discussion

### Principal Findings

In this study, we explored HCPs’ knowledge of secondary uses of health care data and their major concerns about data sharing processes. Participants showed a comprehensive knowledge of the purposes of sharing health care data for purposes beyond individual care, including for uses such as patient profiling, research, quality assurance activities, and public health purposes and to support health care delivery and planning.

Although the majority of the participants mentioned feeling comfortable with data sharing policies and their implementation, they also acknowledged concerns about data sharing, including data accuracy, patients’ willingness to share their records, challenges on obtaining free and informed consent, data security, and potential patient exposure and exploitation.

### Comparison With Previous Work

#### Understanding of Purposes of Sharing Health Care Data for Secondary Uses

The use of patient-level data for population segmentation, tailored care, and understanding patterns in patient needs has been recognized in the literature as one of the biggest promises of big data in health care [21]. Computing and analytics frameworks allow aggregation and integration of big data and the identification of more accurate, stratified disease risk profiles, which can then be targeted with care models and intervention programs tailored to specific population segments’ needs [3].

Similarly, several studies have acknowledged the importance of sharing EHR data for research purposes. In the last decade, the United Kingdom has witnessed a surge in the secondary use of health care data to derive population-based insights, namely, concerning mental health status [22] and prescription patterns [23]. In the United States, pilot studies have already started predicting readmissions and estimating the risk of complications in newborns [21]. Once impossible because of the effort required to collect data, these studies have now become possible by reusing data originally collected for the purposes of individual care, thus maximizing the potential for the use of EHR data.

Previous literature also highlighted the importance of sharing health care data in the context of quality assurance activities [24], which was similarly acknowledged by the participants of this study. It is important to note, however, that these approaches have potential limitations, as EHR-derived measures can undercount practice performance [25].

The use of health care data was also previously suggested to be a powerful tool to support public health activities and facilitate timely and efficient surveillance of both noncommunicable and communicable diseases [4,26].

As acknowledged by our participants, sharing EHR data can also inform strategic planning and service delivery. As previously discussed, strategic planning can be informed by stratification by patients’ risk and also by specific geographic needs and discrepancies. In line with our findings, Rumsfeld et al also suggest that geocoded health care data as a source for big data analytics might improve targeting of community and health resources for patients [2].

#### Challenges of Health Care Data Sharing

Concerns about data accuracy and data quality have been previously addressed in the literature and are critical when extracting real data from the massive, variable, and complex health care datasets, as suboptimal data quality can lead to low utilization efficiency and, importantly, poorly informed decision making [27].

Interestingly, although the participants reported being concerned about patients’ unwillingness to share data, studies assessing patients’ perspectives do not seem to validate this perception. Public members express a widespread willingness to share health care data for secondary uses and, particularly, for research purposes, both in the United Kingdom (68.7%-91.4%) [5,6] and the United States (92%-100%) [7]. Nevertheless, patients’ confidence about sharing data varies greatly depending on ethnicity, social class, and working status, with some remarkable negative perceptions among lower socioeconomic groups and ethnic minorities [28]. Furthermore, although members of the public are generally supportive of data to be shared and used for the purpose of helping others or improving health care, they tend to be less supportive for data to be used by commercial companies [28].

The main concern raised in this study was toward data security and, particularly, about the risk of data breaches, which can lead to patient exposure and possible exploitation. GPs and social care professionals want reassurance that partner organizations with whom they share data are protecting people’s confidential data. Similar concerns were also raised by patients in other studies, including data leakage, loss or reidentification, unauthorized access, and sharing with third parties [29]. However, despite significant recent events (such as the accidental disclosure of a clinic list of email addresses at an HIV clinic [30], the WannaCry malware attack [31], and data sharing without consent in the Pharmacy 2U incident [32]) patients’ confidence levels remain high. Overall, 77% of the public reported trusting the ability of the NHS to protect health care data. The health sector remained the most trusted by the public, ahead of sectors such as retail, banking, and other public services [33].

### Strengths, Limitations, and Future Work

Our purposive sampling strategy ensured that HCPs from a variety of professional roles, health care settings, experience with data sharing, and geographic location were represented. Although we achieved sample diversity, we acknowledge that future surveys using randomized, more powerful samples could explore the determinants of HCPs’ concerns toward patients’ data sharing and assess the external validity of our key findings.

It must also be noted that the understanding and experiences of HCPs toward data sharing for nonindividual care were retrospectively assessed and that several relevant initiatives took place recently. This research temporally overlapped with a new campaign launched by the NHS in England, in partnership with the Information Commissioner’s Office (“Your data matters–building trust and confidence”), aiming to increase the public’s trust and confidence in how their data are both used and made available [34]. Importantly, this initiative provided resources to support health and care staff, including tailored materials for GP practices, nursing, midwifery, and care staff, and opened a register for the ones wanting to publicly pledge their support for their service users’ data rights [34]. In addition, in Europe, the European Union General Data Protection Regulation, in May 2018, has also recently raised awareness, in a helpful way, in professionals and the public on data privacy [35]. We anticipate that longitudinal prospective studies could provide further light on temporal trends on this subject and, importantly, to explore the impact of these recent policies and initiatives on HCP perspective toward EHR data sharing.

### Conclusions and Implications for Policy Makers and Public Communication

Although our results suggest a good understanding of the purposes of data sharing for secondary uses, some concerns still remain. HCPs seem particularly concerned with data accuracy and consent procedures; to mitigate these concerns, they advocate clear policies to appraise and monitor data quality as well as clear procedures to obtain consent. To address concerns related to data security and potential exposure and exploitation of patients’ data, cybersecurity emerges as an important part of the health care culture. To that end, both infrastructure investment and culture change are crucial to minimize accidental or malicious data breaches [31] that can harm individuals and organizations and, importantly, erode both patients’ and HCP’s trust.

HCPs were also concerned about patients’ unwillingness to share data, a perception that does not seem to be corroborated by studies assessing patients’ perspectives [5-7] and that might negatively impact how they communicate the importance of data sharing to patients. Communication policies must provide HCPs with this evidence to reinforce their confidence in patients’ overall receptivity and highlight the importance of targeted communication strategies to improve negative perceptions among lower socioeconomic groups and ethnic minorities.

In the broader context of health care data use for secondary purposes, HCPs are a key partner in creating a patient-centric system across the NHS [34]. A better understanding of their knowledge and concerns can inform national and international communication policies and, importantly, engage them as active contributors to a nuanced decision-making process with and for patients, thus supporting a more widespread learning culture to fully embrace the potential of health care data use.

## Appendix

Multimedia Appendix 1 Topic guide.

Multimedia Appendix 2 Consolidated Criteria for Reporting Qualitative studies (COREQ) checklist.

## Abbreviations

EHR: electronic health record
GP: general practitioner
HCP: health care professional
NHS: National Health Service
NIHR: National Institute for Health Research
