# Supplementary material for: Health Care Professionals’ Perspectives on the Secondary Use of Health Records to Improve Quality and Safety of Care in England: Qualitative Study
Source: J Med Internet Res. 2019 Sep 26;21(9):e14135. doi: 10.2196/14135 (PMC6787532; doi:10.2196/14135)
Supplement: Multimedia Appendix 1 [file jmir_v21i9e14135_app1.pdf]

Bulletin Board Topic Guide

Questions focusing on Knowledge and Understanding of Data Sharing

Understanding of individuals/ organisations
Do you feel that you understand what individuals or organisations have access to this patient-level data?

Understanding of purpose
Do you feel that you understand the purposes for which patients may be asked to share their personal information for reasons for their direct care by other organisations? Beyond their direct care?

Patient data shared
What sort of patient data do you share with other care organisations? How is this communicated to patients, if at all?

Awareness of policies
Please describe any local policies which are in place relating to sharing patient data in your organisation. Please describe any national policies about sharing patient data that you know of.

Learning about policies
How did you learn about your organisation's/national policies about patient-level data sharing? Who provided you with this information? Was it someone within your organisation or outside your organisation?

Experience of concerns (closed)
Have you ever had/ do you currently have concerns about policies around the sharing of your patients' data for secondary use?
