# Supplementary material for: Health Care Professionals’ Perspectives on the Secondary Use of Health Records to Improve Quality and Safety of Care in England: Qualitative Study
Source: J Med Internet Res. 2019 Sep 26;21(9):e14135. doi: 10.2196/14135 (PMC6787532; doi:10.2196/14135)
Supplement: Multimedia Appendix 2 [file jmir_v21i9e14135_app2.pdf]

Manuscript: Secondary use of healthcare data to improve quality and safety of care: healthcare professionals' knowledge and concerns

Consolidated criteria for reporting qualitative studies (COREQ): 32-item checklist

Developed from:
Tong A, Sainsbury P, Craig J. Consolidated criteria for reporting qualitative research (COREQ): a 32-item checklist for interviews and focus groups. *International Journal for Quality in Health Care*. 2007. Volume 19, Number 6: pp. 349 – 357

No. Item	Guide questions/description	Reported Yes (Y) / No (N)
**Domain 1: Research team and reflexivity**		
Personal Characteristics		
1. Interviewer/facilitator	Which author/s conducted the inter view or focus group?	Y
2. Credentials	What were the researcher's credentials? E.g. PhD, MD	Y
3. Occupation	What was their occupation at the time of the study?	Y
4. Gender	Was the researcher male or female?	N
5. Experience and training	What experience or training did the researcher have?	Y
Relationship with participants		
6. Relationship established	Was a relationship established prior to study commencement?	Y
7. Participant knowledge of the interviewer	What did the participants know about the researcher? e.g. personal goals, reasons for doing the research	Y
8. Interviewer characteristics	What characteristics were reported about the inter viewer/facilitator? e.g. Bias, assumptions, reasons and interests in the research topic	Y

**Domain 2: study design**		
Theoretical framework		
9. Methodological orientation and Theory	What methodological orientation was stated to underpin the study? e.g. grounded theory, discourse analysis, ethnography, phenomenology, content analysis	4
Participant selection		
10. Sampling	How were participants selected? e.g. purposive, convenience, consecutive, snowball	Y
11. Method of approach	How were participants approached? e.g. face-to-face, telephone, mail, email	Y
12. Sample size	How many participants were in the study?	Y
13. Non-participation	How many people refused to participate or dropped out? Reasons?	Not available
Setting		
14. Setting of data collection	Where was the data collected? e.g. home, clinic, workplace	Y
15. Presence of non-participants	Was anyone else present besides the participants and researchers?	Y
16. Description of sample	What are the important characteristics of the sample? e.g. demographic data, date	Y
Data collection		
17. Interview guide	Were questions, prompts, guides provided by the authors? Was it pilot tested?	Y
18. Repeat interviews	Were repeat interviews carried out? If yes, how many?	Y
19. Audio/visual recording	Did the research use audio or visual recording to collect the data?	Y
20. Field notes	Were field notes made during and/or after the inter view or focus group?	Not applicable
21. Duration	What was the duration of the interviews or focus group?	Y
22. Data saturation	Was data saturation discussed?	Y
23. Transcripts returned	Were transcripts returned to participants for comment and/or correction?	Y
**Domain 3: analysis and findings**		
Data analysis		

24. Number of data coders	How many data coders coded the data?	Y
25. Description of the coding tree	Did authors provide a description of the coding tree?	Y
26. Derivation of themes	Were themes identified in advance or derived from the data?	Y
27. Software	What software, if applicable, was used to manage the data?	Not applicable
28. Participant checking	Did participants provide feedback on the findings?	Y
Reporting		
29. Quotations presented	Were participant quotations presented to illustrate the themes/findings? Was each quotation identified? e.g. participant number	Y
30. Data and findings consistent	Was there consistency between the data presented and the findings?	Y
31. Clarity of major themes	Were major themes clearly presented in the findings?	Y
32. Clarity of minor themes	Is there a description of diverse cases or discussion of minor themes?	Y
